# Supplementary material for: Unimolecular Micelles from Randomly Grafted Arborescent Copolymers with Different Core Branching Densities: Encapsulation of Doxorubicin and In Vitro Release Study
Source: Materials (Basel). 2023 Mar 20;16(6):2461. doi: 10.3390/ma16062461 (PMC10053569; doi:10.3390/ma16062461)
Supplement: Supplementary file 1 [file materials-16-02461-s001.zip › materials-2269882-supplementary.pdf]

materials

Supporting Information

Unimolecular Micelles from Randomly Grafted Arborescent Copolymers with Different Core Branching Densities: Encapsulation of Doxorubicin and *In Vitro* Release Study

Mosa Alsehli [1,2] and Mario Gauthier [2,*]

[1] Department of Chemistry, Taibah University, Medina P.O. Box 344, Saudi Arabia
[2] Department of Chemistry, Institute for Polymer Research, University of Waterloo, 200 University Ave. W., Waterloo, ON N2L 3G1, Canada
* Correspondence: mario.gauthier@uwaterloo.ca

Synthetic Procedures

Synthesis of γ-Benzyl L-Glutamic Acid *N*-Carboxyanhydride (Glu-NCA)

The monomer was synthesized from γ-benzyl L-glutamic acid and triphosgene as described in the literature [1]. γ-Benzyl L-glutamic acid (Bz-Glu) (10.0 g; 42.0 mmol) was dispersed in 300 mL of freshly distilled ethyl acetate in a 1-L round-bottomed flask fitted with a reflux condenser and a N_2 bubbler. After heating to reflux, triphosgene (4.8 g, 16 mmol) was added at once and refluxing was continued for 3.5 h. The flask was allowed to cool to room temperature, and then stored in a refrigerator (5 ˚C) for 2 h. The cold reaction mixture was transferred to a cold separatory funnel, washed with 100 mL of distilled water chilled to 0 ˚C, and then with 100 mL of chilled 0.5% aqueous $NaHCO_3$ solution. The ethyl acetate layer was dried over anhydrous $MgSO_4$, filtered by gravity, and concentrated to 100–120 mL on a rotary evaporator. An equal volume of cold hexane was then added to induce crystallization of the monomer. After chilling to -5 ˚C overnight, the monomer crystals were

collected by filtration in a Schlenk funnel under N_2. For further purification, in some cases, the monomer was recrystallized twice from *n*-hexane and ethyl acetate (*n*-hexane/ethyl acetate v/v: 2/1), to remove the last traces of remaining impurities. The product was dried under vacuum and stored under N_2 in a freezer until used. Yield = 9.7 g (88 %); ^1H NMR (300 MHz, $CDCl_3$): 7.55–7.22 (s, 5H), 6.72 (s, 1H), 5.09 (s, 2H), 4.35–4.31 (t, 1H), 2.57–2.51 (t, 2H), 2.33–2.19 (m, 1H), 2.18–2.00 (m, 1H).

Synthesis of Poly(γ-benzyl L-glutamate) PBG Serving as Side Chains

A linear polymer serving as side chains was synthesized as described previously [2]. Briefly, Glu-NCA (8 g, 30.4 mmol) was dissolved in dry DMF (20 mL) at 0 °C. *n*-Hexylamine (182 µL, 1.38 mmol, for a target X_n = 22) was then added to the monomer and the reaction was allowed to proceed for 2.5 days at 0 °C. The linear polymer was recovered by precipitation in cold methanol, centrifugation, and drying under vacuum overnight before characterization. Yield = 82%, M_n = 5,100 g/mol, M_w/M_n = 1.09. ^1H NMR (300 MHz, DMSO-d_6): X_n = 22.0, δ: 8.01 (b, 1H), 7.45–7.18 (s, 5H), 5.01–4.88 (s, 2H), 4.09–3.87 (b, 1H), 2.32–1.88 (b, 4H), 1.31–1.15 (b, 0.4H), 0.76–0.74 (s, 0.14H).

Synthesis of Linear PBG Serving as Substrates

Three linear PBG substrates with either 15, 30 or 68 repeating units were synthesized. The number of PBG repeating units was controlled by the amount of Glu-NCA and *n*-hexylamine used in the reaction. The Glu-NCA (4 g, 15.2 mmol) was dissolved in dry DMF (10 mL) and *n*-hexylamine (134 µL, 1.01 mmol, 67 µL, 0.50 mmol and 29.5 µL, 0.22 mmol for target X_n = 15, 30 and 68, respectively) was then added. The reaction was allowed to proceed at room temperature for 3, 4 and 5 h,

respectively, before quenching with acetic anhydride (780 µL) to deactivate the terminal amine moiety. After 1 h the product was precipitated in cold methanol, centrifuged and dried under vacuum overnight before characterization.

The different linear PBG substrates are identified in the form PBG_x, where X denotes the experimental number of PBG repeating units in the chains.

(PBG with target X_n = 15) Yield = 89%, M_w/M_n = 1.17. ^{1}H NMR (300 MHz, DMSO-d_6): δ: 8.01 (b, 1H), 7.45–7.18 (s, 5H), 5.01–4.88 (s, 2H), 4.09–3.87 (b, 1H), 2.32–1.88 (b, 4H), 1.31–1.15 (b, 0.2H), 0.76–0.74 (s, 0.20H). X_n = 15 (PBG_{15})

(PBG with target X_n = 30) Yield = 90%, M_w/M_n = 1.18. ^{1}H NMR (300 MHz, DMSO-d_6): δ: 8.01 (b, 1H), 7.45–7.18 (s, 5H), 5.01–4.88 (s, 2H), 4.09–3.87 (b, 1H), 2.32–1.88 (b, 4H), 1.31–1.15 (b, 0.2H), 0.76–0.74 (s, 0.10H). X_n = 29 (PBG_{29})

(PBG with target X_n = 68) Yield = 92%, M_w/M_n = 1.19. ^{1}H NMR (300 MHz, DMSO-d_6): δ: 8.01 (b, 1H), 7.45–7.18 (s, 5H), 5.01–4.88 (s, 2H), 4.09–3.87 (b, 1H), 2.32–1.88 (b, 4H), 1.31–1.15 (b, 0.2H), 0.76–0.74 (s, 0.05H). X_n = 65 (PBG_{65})

Synthesis of Partially Deprotected Linear PBG Substrates

A linear polymer PBG_{29} (3.05 g, 13.9 mmol Bz-Glu units) was dissolved in a mixture of TFA (33 mL) and 33% (w/w) HBr solution in acetic acid (0.88 mL, 4.17 mmol). The reaction was stirred for 3

h at room temperature before the polymer was precipitated in diethyl ether and recovered by suction filtration to give a product with 31% of free glutamic acid moieties. Yield = 64%.

PBG_{29}-CO_2H. ^1H NMR (300 MHz, DMSO-d_6): δ: 8.01 (b, 1H), 7.45–7.18 (s, 5H), 5.01–4.88 (s, 2H), 4.09–3.87 (b, 1H), 2.32–1.88 (b, 4H), 1.31–1.15 (b, 0.2H), 0.76–0.74 (s, 0.1H). X_n = 29.0

The partially deprotected linear PBG_{65} substrate was synthesized by the same procedure described above to give a product with 31% of free glutamic acid moieties. Yield = 68%.

PBG_{65}-CO_2H. ^1H NMR (300 MHz, DMSO-d_6): δ: 8.01 (b, 1H), 7.45–7.18 (s, 5H), 5.01–4.88 (s, 2H), 4.09–3.87 (b, 1H), 2.32–1.88 (b, 4H), 1.31–1.15 (b, 0.2H), 0.76–0.74 (s, 0.05H). X_n = 65.0

Synthesis of Fully Deprotected Linear PBG Substrate

Sample PBG_{15} (2.90 g, 13.2 mmol Bz-Glu units) was dissolved in a mixture of TFA (30 mL) and 2.8 mL (13 mmol) of 33% (w/w) HBr solution in acetic acid. The reaction was allowed to stir for 3 h at room temperature before the polymer was precipitated in diethyl ether. After drying under vacuum the precipitate was dissolved in and dialyzed against distilled water, and subsequently freeze-dried, yielding a polymer with fully deprotected glutamic acid moieties as a white solid. Yield = 43%.

PBG_{15}-CO_2H. ^1H NMR (300 MHz, DMSO-d_6): δ: 8.01 (b, 1H), 4.09–3.87 (b, 1H), 2.32–1.88 (b, 4H), 1.31–1.15 (b, 0.2H), 0.76–0.74 (s, 0.2H). X_n = 15.0

Synthesis of G1 and G2 Arborescent PBG

Briefly, G0PBG (2.2 g, 10.0 mmol Bz-Glu units) was first partially deprotected by the procedure described above, and recovered in 53% yield with 31 mol % of free glutamic acid moieties. In a typical coupling reaction for the synthesis of G1PBG, the partially deprotected G0PBG substrate (0.3 g, 0.48 mmol -CO_2H) and the polymer serving as side chains (2.27 g, 0.44 mmol chains) were dissolved in 15 mL of dry DMSO. The peptide coupling reagents DIC (0.42 mL, 2.7 mmol) and HOBt (0.36 g, 2.7 mmol) were then added to the reaction with TEA (0.31 mL, 2.2 mmol). The reaction was allowed to proceed for 36 h at room temperature before adding *n*-hexylamine (0.32 mL, 3.2 mmol) to deactivate residual carboxylic acid sites. After 3 h the product was recovered by precipitation in cold methanol, suction filtration, and drying under vacuum. Unreacted side chains were removed from the crude polymer by preparative SEC. The G2 arborescent PBG samples were synthesized and purified as described for the G1 sample.

Quantification of Primary Amine Functionality Level by ^{19}F NMR Analysis

^{19}F NMR spectroscopy was used to determine the chain end primary amine functionality level, f_{NH2}, of the linear polymers used in the grafting reactions as described in the literature [3]. The PBG sample (0.10 g, 1.72×10^{-5} mol chains) was first dissolved in 3 mL of deuterated DMSO (DMSO-d_6). A solution of 4-trifluoromethylbenzaldehyde (TFBA, 0.0901 g, 5.17×10^{-4} mol) and benzotrifluoride (BTF, 0.0756 g, 6.16×10^{-4} mol) in 2 g of DMSO-d_6 was then prepared, and 0.1706 g of that reagent solution (4.563×10^{-5} mol TFBA, 4.531×10^{-5} mol BTF) was added to the PBG solution which was stirred for 2 h before it was transferred to an NMR tube for analysis.

Synthesis of Diphenylmethylpotassium

Diphenylmethylpotassium (DPMK) was prepared as described in the literature [4]. Briefly, in a flame-dried 3-neck round-bottom flask (RBF) with a magnetic stirring bar, attached to a high-vacuum line, 150 mL of dry THF was introduced followed by potassium metal (4.2 g, 107 mmol, 2 eq) cut into small pieces. Naphthalene (6.89 g, 53.7 mmol, 1 eq) was added, at which point the solution turned dark green. After stirring for 30 min, diphenylmethane (18.1 mL, 107 mmol, 2 eq) was added to the flask with a syringe. All these additions were performed under nitrogen atmosphere. The reaction was allowed to proceed overnight to give a dark red DPMK solution.

Determination of the Concentration of the DPMK Solution

In a flame-dried 3-neck RBF with a magnetic stirring bar and purged with nitrogen, attached to a high-vacuum line, 30 mL of dry THF were introduced followed by a few drops of DPMK solution, until the solution retained a pale yellow coloration. Acetanilide (50 mg, 0.37 mmol) was then added, at which point the solution became colorless. The DPMK solution was then slowly added (0.75 mL) to obtain the same pale yellow color present initially. The concentration of DPMK was determined to be 0.49 M.

Ethylene Oxide Purification

Ethylene oxide (EO) is very toxic in both its liquid and gas forms, so it must be handled with special care in a well-ventilated fume hood. A vacuum manifold was connected to the EO tank line, and a thick-wall (double-thickness) ampoule with a PTFE stopcock, a magnetic stirring bar, and approximately 2 g of calcium hydride as drying agent. The apparatus was thoroughly flamed to

remove adsorbed water before the manifold was sealed, the ampoule was cooled in liquid nitrogen, and EO was introduced slowly and condensed in the ampoule. The ampoule was then removed and mounted on a vacuum manifold with a RBF containing a magnetic stirring bar and another ampoule with a PTFE stopcock. The EO was degassed with three successive freeze-pump-thaw cycles. After closing the ampoule containing the EO the rest of the manifold was evacuated, flame-dried and purged with nitrogen. Then the phenylmagnesium chloride solution (PhMgCl, 10 mL, 2.0 M in THF) was added to the RBF. After removing the THF under vacuum the EO was recondensed to the RBF, stirred with the PhMgCl in an ice bath for 1 h, and slowly recondensed to the empty ampoule, which was subsequently removed and stored in the fume hood.

Polymerization of EO

Poly(ethylene oxide) (PEO) with a primary amine end group was synthesized using a vacuum manifold with a 5-neck RBF containing a magnetic stirring bar and the sealed ampoule containing the purified EO monomer (17.4 g, 0.395 mol). After the RBF was flame-dried under vacuum and purged with nitrogen, dry THF (200 mL) was added. The DPMK solution was introduced drop-wise through one neck until a faint yellow color persisted, followed by 3-aminopropanol (0.138 mL, 1.75 mmol). Additional DPMK solution (4.7 mL, 2.3 mmol) was then added to deprotonate the alcohol. The EO monomer (17.4 g, 0.395 mol, for a target $X_n = 226$, $M_n = 10000$ g/mol) was added to the reaction, transferred to the ampoule and sealed carefully. The reaction was allowed to proceed for 9 days at 45 °C in an oil bath, after which acidified methanol (0.5 mL, 1/10 v/v HCl/methanol) was added to the dark brown solution to terminate the reaction. The solution was concentrated by rotary

evaporation and precipitated in diethyl ether. The brownish powder recovered by filtration was redissolved in methanol and precipitated again in diethyl ether. The polymer, recovered by suction filtration and drying under vacuum, was obtained as a white powder. Yield: 12.8 g (73%). SEC (THF): M_n^{app} = 10,100 g/mol, M_w/M_n = 1.12. ^1H NMR: (300 MHz, $CDCl_3$): δ: 3.87-3.35 (m, 912H), 3.1 (br, 2H), 1.96 (br, -OH), X_n = 228 (M_n = 10,100 g/mol).

Table S1. Synthesis of linear PBG substrates with different molecular weights.

Sample	Target X_n	^1H NMR X_n	$M_n^{H\ NMR}$	M_n^{app}(SEC)	PDI
PBG_{15}	15	15	3,600	3,880	1.11
PBG_{29}	30	29	6,600	6,900	1.10
PBG_{65}	68	65	14,500	15,700	1.12

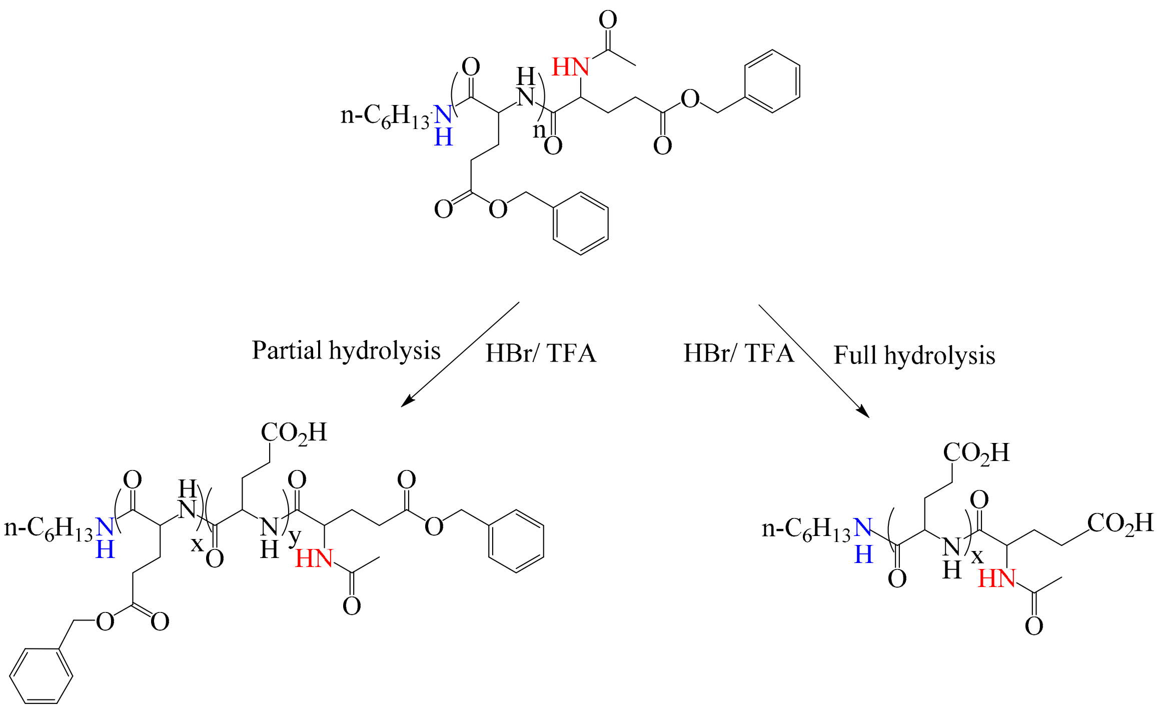

Scheme S1. Partial and complete deprotection of the PBG substrates.

Table S2. Deprotected linear PBG serving as substrates.

Sample	Target X_n	^{1}H NMR X_n	Target deprotection level (%)	^{1}H NMR deprotection level (%)
PBG_{15}	15	15	100	100
PBG_{29}	30	29	30	31
PBG_{65}	68	65	30	31

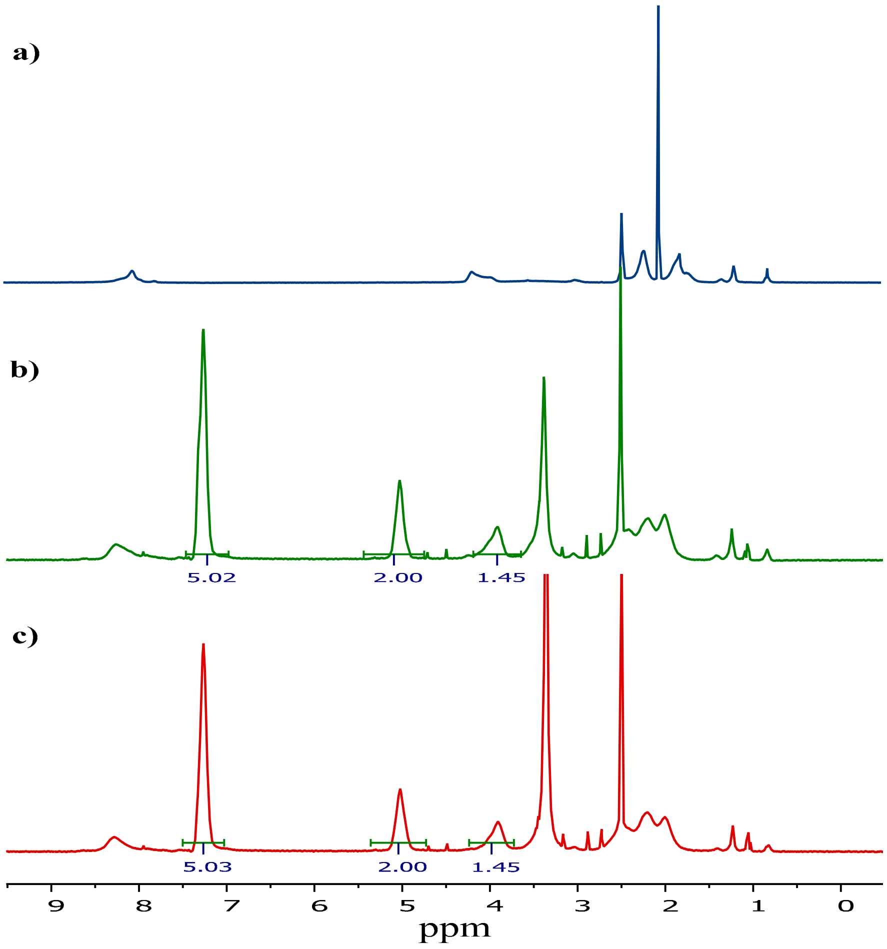

Figure S1. ^{1}H NMR spectra for (a) fully deprotected linear PBG_{15}, (b) partially deprotected linear PBG_{29}, and (c) partially deprotected linear PBG_{65}.

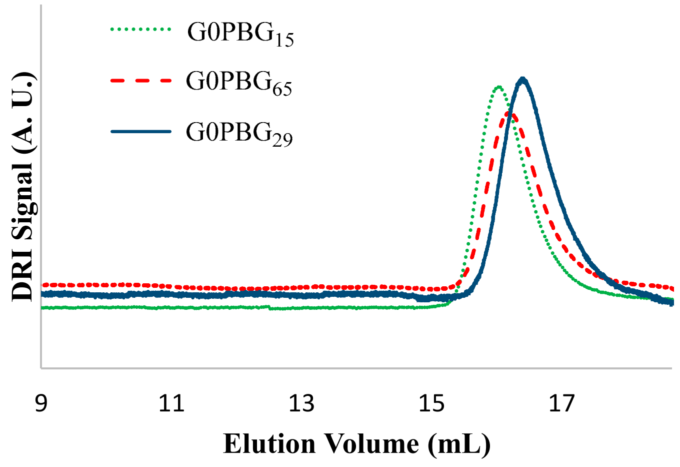

Figure S2. SEC traces for G0 arborescent PBG samples.

Table S3. Characteristics of upper generation PBG arborescent substrates.

Sample	M_n (g/mol)[a]	M_w/M_n[a]	G_y (%)[b]	C_e (%)[c]	f_n[d]
$G1PBG_{15}$	240,000	1.07	31	39	35
$G1PBG_{29}$	200,000	1.06	54	66	32
$G1PBG_{65}$	260,000	1.09	53	60	42
$G2PBG_{15}$	1.2×10^6	1.09	50	59	192
$G2PBG_{29}$	1.0×10^6	1.08	47	57	156
$G2PBG_{65}$	1.4×10^6	1.05	56	64	228

[a] Values from SEC-MALLS analysis in DMSO; [b] Grafting yield: fraction of side chains attached to the substrate; [c] Coupling efficiency: fraction of coupling sites on the substrate consumed in the reaction; [d] Branching functionality: number of side chains added in the last grafting cycle.

Synthesis of Linear Amine-terminated Poly(ethylene oxide)

Poly(ethylene oxide) with a primary amine terminus was used as hydrophilic shell material to demonstrate the synthesis of water-dispersible arborescent copolymer micelles. The anionic polymerization of ethylene oxide with a bifunctional initiator, 3-aminopropanol deprotonated with diphenylmethylpotassium (DPMK), was used to obtain linear PEO with a primary amine chain end as shown in Scheme S2.

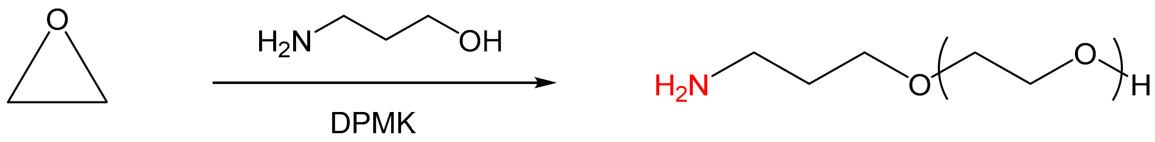

Scheme S2. Polymerization of ethylene oxide with 3-aminopropanol and DPMK.

The ^{1}H NMR spectrum obtained for the α-amino PEO sample synthesized is shown in Figure S3. The ratio of intensities for the -CH_2- protons next to the terminal amine (δ 3.1 ppm) and the four protons in the repeat units (δ 3.6 ppm) was used to determine the number-average degree of polymerization X_n = 228, which corresponds to M_n = 10,100 g/mol.

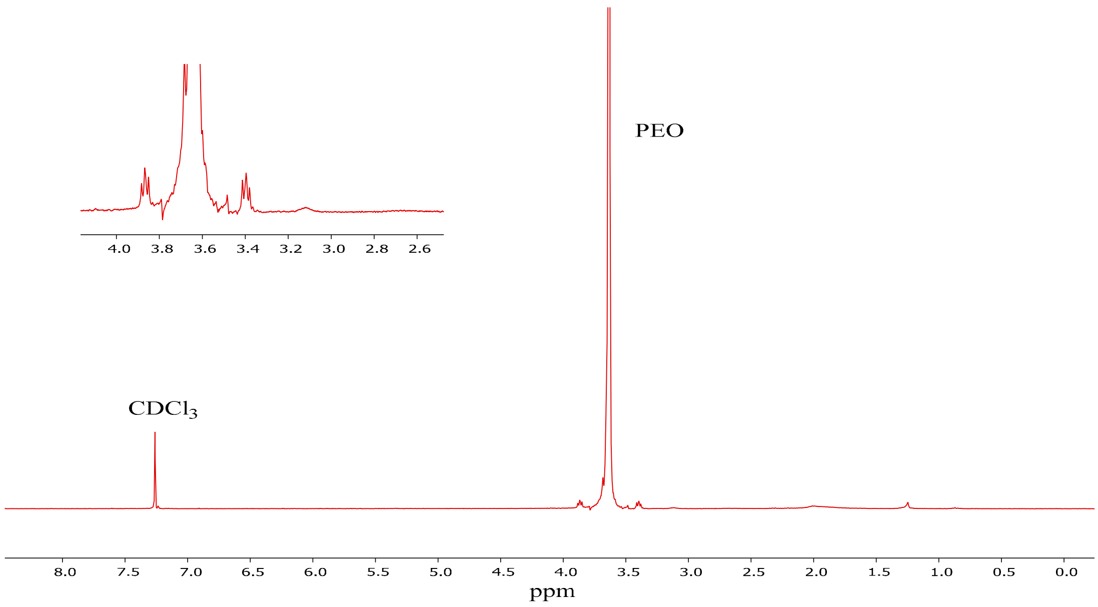

Figure S3. ^{1}H NMR spectrum for α-amino PEO in $CDCl_3$.

^{19}F NMR analysis served to determine the amine functionality level of PEO (f_{NH2}). The absence of primary amine end groups would lead to linear PEO chains being incapable of coupling with the substrate. A procedure developed by Ji et al. [3] was used to determine the primary amine functionality level of the PEO side chains. The terminal amine was reacted with 4-trifluoromethylbenzaldehyde (TFBA) to produce imine functionalities at the chain end of the polymer as shown in Scheme S3. ^{19}F NMR spectroscopy was then employed to observe the chemical shift of the fluorine atoms in the imine formed, to be compared with the signal for benzotrifluoride (BTF) serving as internal standard.

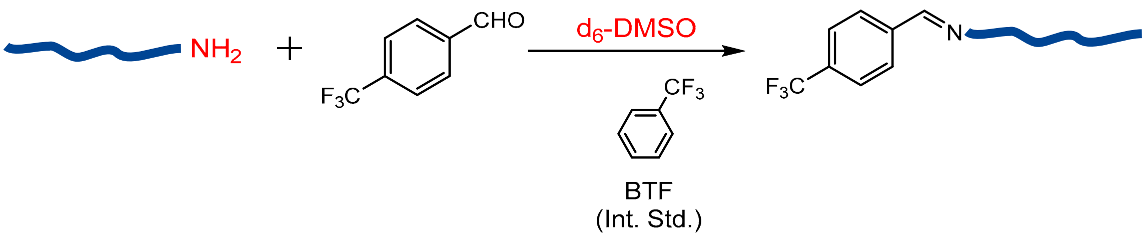

Scheme S3. Reaction of 4-trifluoromethylbenzaldehyde (TFBA) with the primary amines end group in the PEO side chains.

The ^{19}F NMR spectrum obtained for the linear PEO sample synthesized using the 3-aminopropanol/DPMK initiator system is shown in Figure S4. The amine functionality level f_{NH2} of the PEO was calculated by integrating the peaks in the ^{19}F NMR spectrum. The number of moles of chains present in the reaction was determine by comparing the integration value for the imine fluorines (0.346) to the integration value for the internal standard BTF fluorides (1.000). An amine functionality level f_{NH2} = 94% was thus obtained.

$$f_{NH2} = \frac{mol\ imine}{mol\ polymer\ chains} = \frac{mol\ BTF \times \left(\frac{Imine\ integration}{BTF\ integration}\right)}{\left(\frac{g\ of\ polymer}{M_n\ of\ polymer}\right)} = \frac{2.69 \times 10^{-5}\ mol \times \left(\frac{0.346}{1.00}\right)}{\frac{0.100\ g}{10100\ g/mol}} = 0.94 \qquad (S1)$$

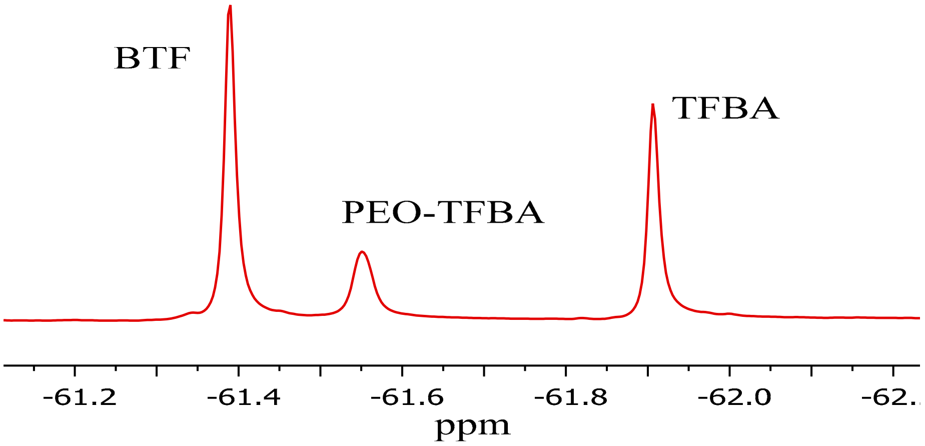

Figure S4. ^{19}F NMR spectrum for linear PEO synthesized using the 3-aminopropanol/DPMK initiator system, after treatment with 4-trifluoromethylbenzaldehyde (TFBA).

Calculation of the Grafting Yield and Branching Functionality for G1PBG15-*g*-PEO as an Example

In this reaction 0.59 g of PEO side chains and 0.04 g of PBG substrate (corresponding to a NH_2/CO_2H ratio of 0.90) were used. Weight fractions for PEO and PBG of 0.80 and 0.20, respectively, were determined when comparing the molecular weights of the copolymer and the substrate. Multiplying the known mass of PBG used in the grafting reaction (0.04 g) by the weight fraction ratios (0.8/0.2) yields a mass of 0.16 g PEO in the copolymer. Dividing this by the total mass used in the grafting reaction (0.59 g PEO), a grafting yield of 27% was obtained [5].

$$G_y = \frac{mass\ PBG\ (g) \times \frac{Wt.Fraction\ PEO}{Wt.Fraction\ PBG}}{mass\ PEO\ (g)} = \frac{0.04 \times \frac{0.8}{0.2}}{0.59} = 0.27 \qquad \text{(S2)}$$

The weight fraction of PEO in the copolymers was determined by dividing the increase in molecular weight for the copolymers by their total molecular weight.

$$f_\mathrm{n} = \frac{M_n(G1PBG_{15}\text{-}g\text{-}PEO) - M_n(G1PBG_{15})}{M_n(PEO)} = \frac{1{,}200{,}000 - 240{,}000}{10{,}000} = 96 \qquad \text{(S3)}$$

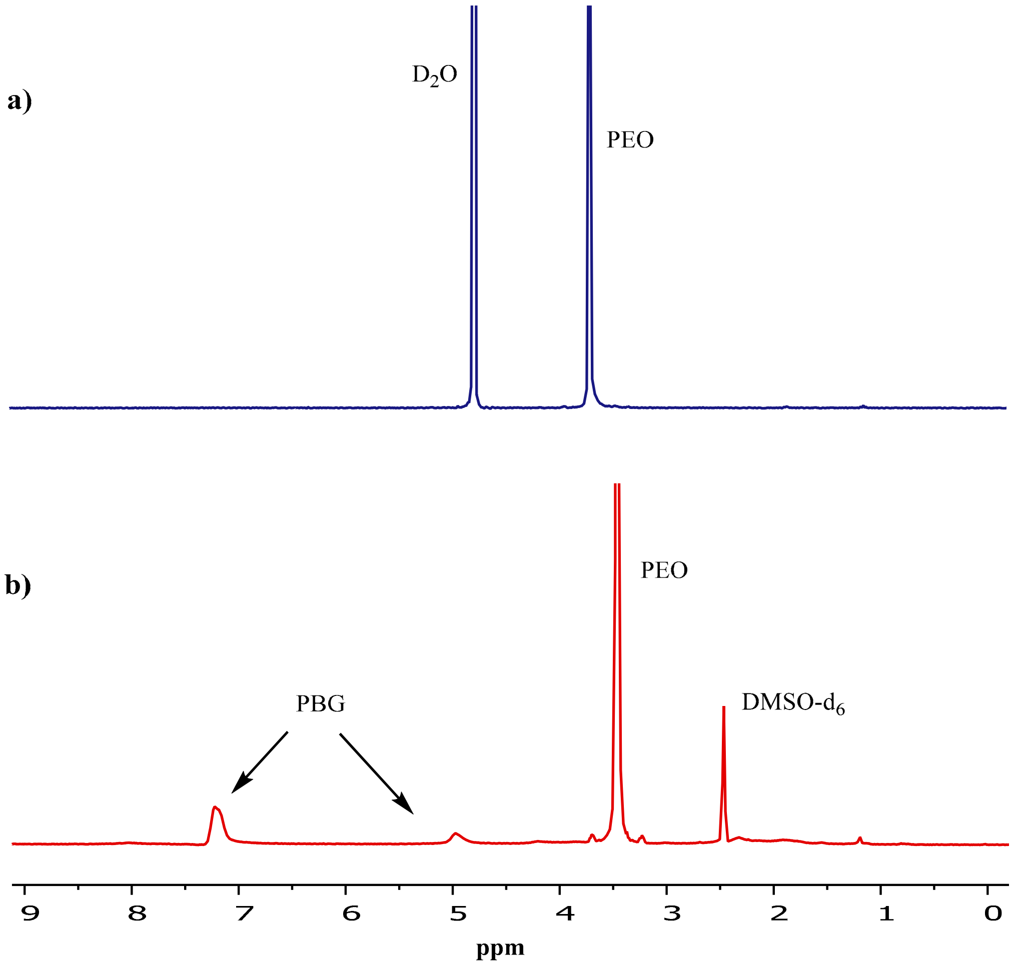

Figure S5. ^{1}H NMR spectra for G2PBG29-g-PEO in (a) D2O and (b) deuterated DMSO.

Table S4. Characteristics of randomly grafted PBG-*g*-PEO arborescent copolymers.

Copolymer	DMF				PBS			
	$D_{h,number}$	$D_{h,volume}$	$D_{h,intensity}$	PDI	$D_{h,number}$	$D_{h,volume}$	$D_{h,intensity}$	PDI
G1PBG$_{15}$-*g*-PEO	16 ± 1	18 ± 1	21 ± 1	0.18	16 ± 1	19 ± 1	22 ± 1	0.41
G1PBG$_{29}$-*g*-PEO	15 ± 1	18 ± 1	22 ± 1	0.16	17± 2	22 ± 2	27 ± 2	0.50
G1PBG$_{65}$-*g*-PEO	18 ± 1	22 ± 1	26 ± 1	0.09	19 ± 2	23 ± 2	28 ± 2	0.43
G2PBG$_{15}$-*g*-PEO	33 ± 2	36 ± 2	43 ± 2	0.06	35 ± 2	40 ± 3	47 ± 2	0.28
G2PBG$_{29}$-*g*-PEO	32 ± 2	36 ± 2	42 ± 2	0.03	34 ± 1	40 ± 1	46 ± 2	0.26
G2PBG$_{65}$-*g*-PEO	37 ± 1	45 ± 1	58 ± 2	0.07	39 ± 1	47 ± 1	58 ± 2	0.17

All experiments done in triplicate. The results are presented as the mean ± standard deviation.

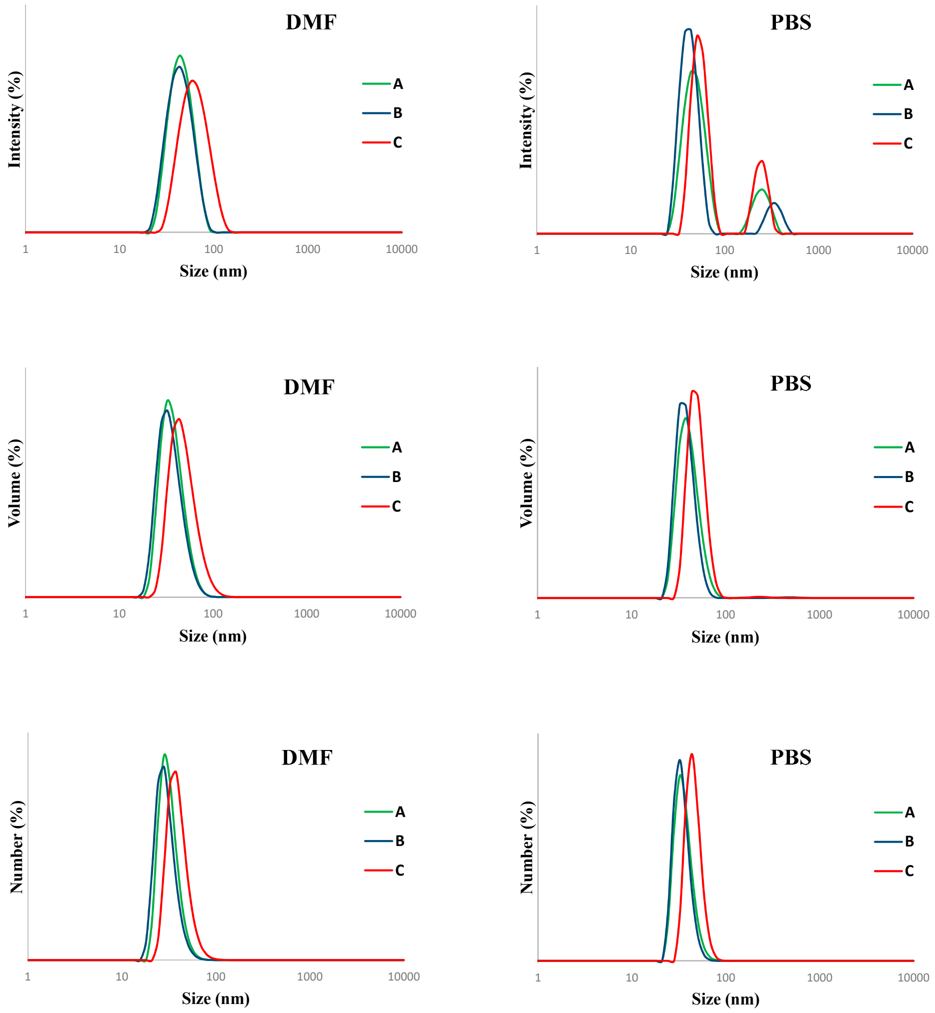

Figure S6. Hydrodynamic diameter distributions for randomly grafted arborescent copolymers determined by DLS in DMF (left) and in PBS (right): (A) G2PBG$_{15}$-*g*-PEO, (B) G2PBG$_{29}$-*g*-PEO, and (C) G2PBG$_{65}$-*g*-PEO.

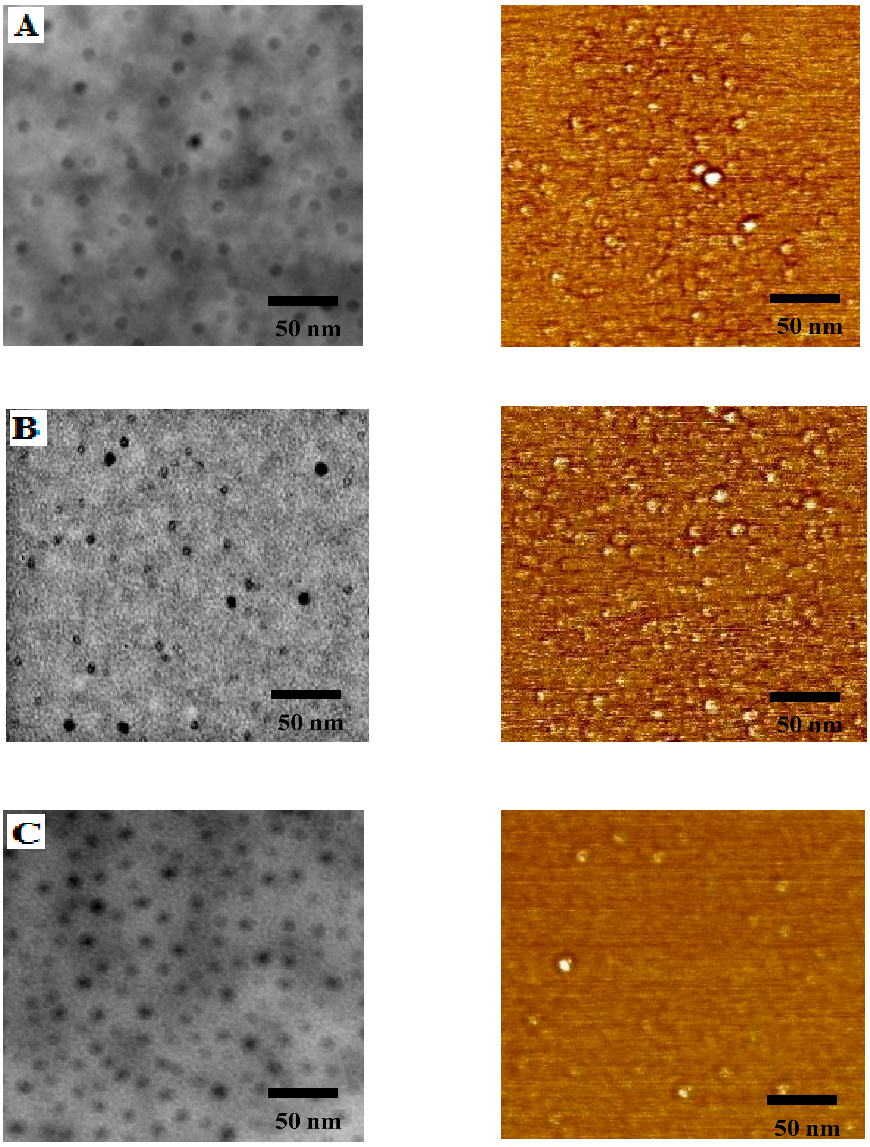

Figure S7. TEM (left) and AFM phase scan (right) for randomly grafted arborescent copolymers: (A) G1PBG$_{15}$-*g*-PEO, (B) G1PBG$_{29}$-*g*-PEO and (C) G1PBG$_{65}$-*g*-PEO.

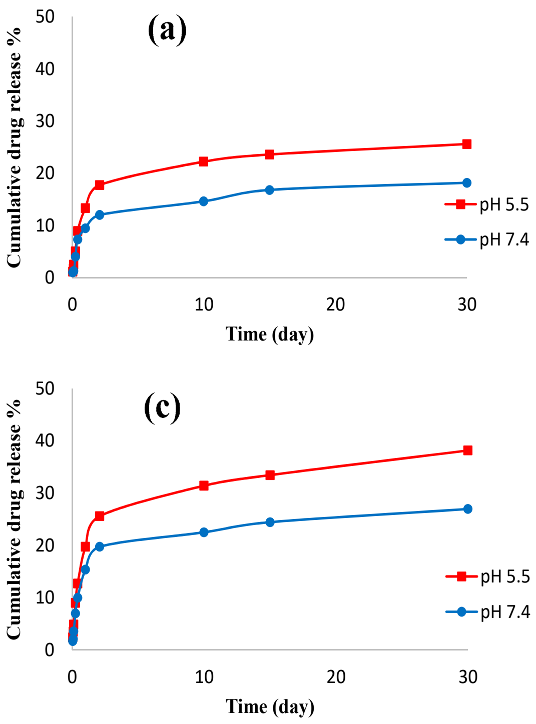

Figure S8. *In vitro* DOX release profiles over a 30-day period for (a) G2PBG15-*g*-PEO/DOX, (b) G2PBG29-*g*-PEO/DOX and (c) G2PBG65-*g*-PEO/DOX in PBS (pH 7.4 and 5.5) at 37 °C.

References

1. Poché, D.S.; Moore, M.J.; Bowles, J.L. An unconventional method for purifying the N-carboxyanhydride derivatives of γ-alkyl-L-glutamates. *Synthetic Communications* **1999**, *29*, 843-854, doi:10.1080/00397919908086042.
2. Whitton, G.; Gauthier, M. Arborescent polypeptides from γ-benzyl L-glutamic acid. *Journal of Polymer Science Part A: Polymer Chemistry* **2013**, *51*, 5270-5279, doi:10.1002/pola.26958.
3. Ji, S.; Hoye, T.R.; Macosko, C.W. Primary amine (−NH_2) quantification in polymers: Functionality by ^{19}F NMR spectroscopy. *Macromolecules* **2005**, *38*, 4679-4686, doi:10.1021/ma050178b.
4. Normant, H.; Angelo, B. Sodation en milieu tétrahydrofuranne par le sodium en présence de naphthalène. *Bulletin De La Societe Chimique De France* **1960**, 354-359.
5. Whitton, G.; Gauthier, M. Arborescent micelles: Dendritic poly(γ-benzyl L-glutamate) cores grafted with hydrophilic chain segments. *Journal of Polymer Science Part A: Polymer Chemistry* **2016**, *54*, 1197-1209, doi:10.1002/pola.27943.
